# Confidence of Pediatric Primary Care Clinicians in Autism Screener Score and Their Own Diagnostic Impressions

**DOI:** 10.3390/bs16020289

**Published:** 2026-02-17

**Authors:** Georgina Perez Liz, Andrea Trubanova Wieckowski, Autumn Austin, Alexia Faith Dickerson, Erika Frick, Ashley Dubin, Ashley de Marchena, Diana L. Robins

**Affiliations:** A.J. Drexel Autism Institute, Dornsife School of Public Health, Drexel University, 3020 Market St. Suite 560, Philadelphia, PA 19104, USAahd45@drexel.edu (A.D.); dlr76@drexel.edu (D.L.R.)

**Keywords:** autism spectrum disorder, early detection, diagnostic impression, toddlers, primary care

## Abstract

Autism-specific screening and developmental surveillance in primary care aid identification of autism. In this study, we assessed primary care clinicians’ (PCCs’) reported confidence in screening scores from the Modified Checklist for Autism in Toddlers, Revised (M-CHAT-R) and in their own diagnostic impressions. Four PCCs provided data for 50 children aged 16–36 months for whom they had any developmental concern. PCCs’ diagnostic impressions were “Definitely Autism” for 15 children (30%), “Unsure—Needs Further Evaluation” for 25 children (50%) and “Definitely Not Autism” for 10 children (20%). They reported High Confidence on the screener score in 33 cases (66%). Of the 17 cases for whom PCCs reported having Low Confidence on the M-CHAT-R, 14 children (82.3%) had a Low Likelihood score, with no significant association between M-CHAT-R likelihood and PCC’s confidence in the screening score. PCCs’ diagnostic impressions were concordant with the M-CHAT-R autism likelihood in 42% of cases, with a significantly higher mean in confidence rating when compared to the non-concordant cases. Language development and social engagement were the most frequently endorsed concerns by PCCs, with significant associations between these concerns and M-CHAT-R likelihood. Our results suggest that, when developmental concerns exist, PCCs may place greater confidence in the M-CHAT-R when scores indicate a higher likelihood of autism, and that confidence in their own diagnostic impressions may be associated with concordance with the screener score.

## 1. Introduction

Autism is characterized by persistent deficits in social communication and social interaction across multiple contexts, and restricted, repetitive patterns of behavior, interests, or activities (APA, 2022). The estimated prevalence of autism in the United States is approximately 1 in 31 8-year-old children (Shaw et al., 2025) and roughly 1 in 100 worldwide (Zeidan et al., 2022). Parents commonly report noticing early symptoms of autism by about two years of age (Leach et al., 2025; Ozonoff et al., 2009); however, the average age of formal diagnosis remains close to four years (Shaw et al., 2025). This persistent diagnostic delay occurs for multiple reasons, including long waiting lists—often lasting a year or more—for evaluation by autism specialists after referral from primary care (Mandell et al., 2005; Rutherford et al., 2018). Additional barriers include workforce shortages, limited availability of developmental–behavioral pediatricians, variations in insurance coverage, and socioeconomic or cultural factors that influence families’ help-seeking behaviors (Aylward et al., 2021). Collectively, these barriers contribute to disparities in access to timely diagnosis and intervention, particularly among children from historically marginalized backgrounds.

The American Academy of Pediatrics (AAP; Hyman et al., 2020) recommends universal autism-specific screening during well-child visits, and advises pediatric clinicians to initiate simultaneous referrals for a comprehensive diagnostic evaluation and autism-specific early intervention (EI) or school-based services when autism concerns arise. This parallel referral model is intended to minimize delays in access to services during a critical period of early neurodevelopment. However, despite clear guidance, such coordination is inconsistently implemented in routine practice, due in part to systemic barriers such as long waitlists for specialty evaluations and fragmented service systems (Rutherford et al., 2018). Additionally, it is worth noting the difference between a diagnosis, as established in the healthcare system, and an educational classification, granted in the educational system. While both allow individuals to receive needed supports, the diagnosis by a healthcare professional relies on the endorsement of current clinical diagnostic criteria by a trained healthcare professional. An educational classification, on the other hand, is a school-based determination under laws like the Individuals with Disabilities Education Act in the United States (IDEA, 2004) based on the impact that a condition may have on a student’s learning ability, and the need to receive special education services and supports (i.e., an Individualized Educational Plan in the United States). As a result, many children who lack a formal medical diagnosis of autism end up receiving only low-intensity, generalized EI services rather than the evidence-based, intensive, autism-specific programs shown to optimize long-term developmental outcomes (Reichow et al., 2018).

Empowering primary care clinicians (PCCs), including pediatricians, family medicine physicians, nurse practitioners, and physician assistants, to diagnose autism could accelerate access to intervention and mitigate disparities. A recent study reported that although the likelihood of children being diagnosed by PCCs has decreased over time, those diagnosed in primary care settings tend to receive their diagnosis approximately one year earlier than children diagnosed by specialists (Smith et al., 2024). The most recent AAP clinical report for autism affirms that general pediatricians comfortable with the application of the DSM-5 criteria can make an initial clinical diagnosis (Hyman et al., 2020). There are several types of behavior that lead PCCs to express developmental concerns for a very young child, including the two domains that form the diagnostic criteria for autism: social engagement and restricted/repetitive behavior, as well as language development. Although delay in language development is not per se a core feature of autism, its common association with autism and its salience for parents when they report concerns makes it a frequent concern expressed for children who go on to receive an autism diagnosis (e.g., Nitzan et al., 2023; Richards et al., 2016).

PCCs may not be able to diagnose all autism cases in young children, but they can likely make accurate diagnostic decisions for a subset of clear-cut presentations that lack the complexity requiring specialist comprehensive assessment (de Marchena & Miller, 2017). Early diagnosis in these cases may allow for prompt intervention and supports, avoiding delays imposed by the traditional referral system and freeing specialist capacity for children with more complex presentations.

Several studies have examined the diagnostic accuracy of PCCs without extensive specialty training. Penner et al. (2023) found that 17 general pediatricians in Canada agreed with a multidisciplinary team 89% of the time when they classified a child as having autism, with only one false-positive case. Similarly, Ahlers et al. (2019) reported strong concordance between general pediatricians and specialist teams in a pilot diagnostic clinic, supporting the feasibility of PCC-led diagnosis. In a recent study by our team, PCCs who classified a child as “definitely having autism” were accurate in 100% of cases, though accuracy was lower (57%) when classifying children as “definitely not having autism” (Wieckowski et al., 2025). Confidence in diagnostic impressions, understood as a cognitive judgment by which PCCs determine how certain they are about their diagnostic impression, appears to relate closely to accuracy. Penner et al. (2023) showed that PCCs’ confidence in an autism diagnostic impression predicted agreement with multidisciplinary teams.

Universal toddler screening for autism is a widely used strategy to support the early detection of autism in primary care. The most commonly used tool is the Modified Checklist for Autism in Toddlers, Revised, with Follow-Up (Robins et al., 2009, 2014), a two-stage tool validated for children aged 16 to 30 months. The first stage, M-CHAT-R, is a 20-item yes/no parent questionnaire with high sensitivity for autism. To reduce false positives, it is recommended by the M-CHAT-R authors to administer the second stage (Follow-Up) when scores fall in the moderate likelihood of autism range (3 to 7). In settings where the Follow-Up is not utilized, any score of 3 or greater is considered High Likelihood of autism.

Although prior studies have examined the diagnostic accuracy and general confidence in identifying autism by clinicians of different backgrounds including pediatric primary care (e.g., Hedley et al., 2016; McNally Keehn et al., 2023; Penner et al., 2023), few have specifically investigated clinicians’ confidence in screening results, such as the M-CHAT-R, and whether or not this confidence differs by likelihood of autism for children for whom there is already a developmental concern, since this may be a factor influencing referrals to diagnostic assessment and intervention. A study by Rosenbaum and Gabrielsen (2019) shows that PCCs’ decision-making factors to refer a child for an autism assessment include items that are addressed by screening tools, such as appropriate play, interactions and engagement, and eye contact. The existing research primarily focuses on the psychometric properties and implementation of the M-CHAT-R/F in different populations and settings, leaving limited understanding of clinicians’ confidence in the screener itself. The present study addresses this gap by evaluating primary care clinicians’ confidence in both their diagnostic impressions and in the M-CHAT-R scores during well-child visits.

The current pilot study builds on our team’s prior work comparing diagnostic impressions formed by primary care clinicians and experts conducting telemedicine assessments with expert diagnosis from in-person evaluations (Wieckowski et al., 2025). Here, we extend that work by exploring clinicians’ confidence in their patients’ autism screening results, and whether or not the concordance between screening outcomes and their diagnostic impressions relates to the confidence rating for their diagnostic impressions, in a sample of children for whom the PCC had developmental concerns. We also explored which aspects of development are reported as concerning in these children by PCCs. Understanding these processes may help identify factors that influence diagnostic decision-making and inform strategies to improve early autism identification in primary care.

## 2. Materials and Methods

Data for this study were drawn from a project conducted by our team on autism diagnostic impressions in primary care and telemedicine contexts (Wieckowski et al., 2025). The current analysis focuses on the subset of data collected from primary care clinicians, specifically examining their confidence ratings in diagnostic impressions and in the M-CHAT-R screening results during well-child visits. This study was approved by the university’s Institutional Review Board. All participating clinicians and parents provided informed consent.

### 2.1. Participants

Four pediatric PCCs (three pediatricians and one physician assistant) from an urban pediatric practice participated. Clinicians were all female; no other demographics were collected from PCCs. PCCs had no specific autism training prior to the study, although one PCC reported attending a continuing education session about early detection of autism in the past. The clinic’s patient population is approximately 70% White, 12% Black/African American, 10% Latinx, 5% multiracial, and 2% Asian, with 15% of families insured through Medicaid.

Out of 67 toddlers enrolled in the parent study, 7 were excluded from the current sample because they were younger than 16 months or older than 36 months (i.e., out of age range). An additional 10 children were excluded because they were missing M-CHAT-R. There was no significant difference in the proportion of Low vs. High Likelihood between the group of children included in the study and those excluded due to age (i.e., younger than 16 months or older than 36 months). Therefore, 50 toddlers were included in the current pilot study. Constraints on funding and project duration dictated the final sample size. The overall mean age of our sample was 30.4 months (*SD* = 5.4). The mean age for the group of children with Low Likelihood M-CHAT-R was 30.7 months (*SD* = 6.8), and the mean age for the group with High Likelihood M-CHAT-R was 28.5 months (*SD* = 3.5), *p* = 0.242. It is important to note that PCCs referred children for whom they had non-autism developmental concerns, in addition to those for whom they had autism concerns. This strategy was employed to reduce bias from the telehealth and in-person clinicians who confirmed diagnosis, so there would not be assumptions about the likelihood of autism.

### 2.2. Measures

Modified Checklist for Autism in Toddlers, Revised (M-CHAT-R; Robins et al., 2009, 2014): The M-CHAT-R is the most widely used toddler autism screening tool, validated for children aged 16 to 30 months. Although the M-CHAT(-R/F) was initially validated for children between 16 and 30 months of age, it has been used with children up to 48 months of age (e.g., Beacham et al., 2018; Christopher et al., 2021). It consists of 20 items with “Yes” or “No” responses for parents to endorse their child’s usual behaviors. A score of 0–2 is considered Low Likelihood of autism and no further action is recommended, whereas a score of 8 or higher is considered High Likelihood of an autism diagnosis and warrants immediate referral to a diagnostic evaluation and early intervention. Screeners with a score of 3–7 are considered Intermediate Likelihood and the recommendation is to administer the Follow-Up to determine recommendation for diagnostic evaluation. However, if the Follow-Up is not utilized, scores of 3 and above indicate a positive screen, especially in children for whom there already are developmental concerns (e.g., Beacham et al., 2018). The pediatric practice routinely administers the M-CHAT-R to parents of toddlers during well-child visits.

Diagnostic Impression Form (DIF): For each eligible child, PCCs completed the DIF, reporting (a) their diagnostic impression (Definitely Autism; Definitely Not Autism; or Unsure—Needs Further Evaluation); (b) their confidence in that impression on a 5-point Likert scale (1 = Not Very Confident; 3 = Confident; 5 = Extremely Confident); (c) their confidence in the M-CHAT-R score on a 5-point Likert scale (1 = Not Very Confident; 3 = Confident; 5 = Extremely Confident); and (d) the behavioral concerns leading to their diagnostic impression (i.e., social engagement, language, restricted/repetitive behavior, or other). Social engagement and restricted/repetitive behavior were included because they are the key domains of autism symptoms; language and Other were included to capture other common types of concerns PCCs have for children with possible autism or other developmental delay.

### 2.3. Procedures

PCCs completed the DIF and reported the M-CHAT-R scores through an online Qualtrics survey during routine well-child visits for any eligible child for whom they had developmental concerns. PCCs rated their confidence in the screener scores and in their own diagnostic impressions after they had access to see the M-CHAT-R scores. As part of the main study, families were invited to telehealth and in-person diagnostic assessments, with diagnostic outcome data reported elsewhere (Wieckowski et al., 2025).

### 2.4. Statistical Analysis

For this study, we categorized M-CHAT-R scores into Low Likelihood (scores of 0 to 2) and High Likelihood (scores of 3 to 20); see Measures for rationale. PCCs’ confidence in the M-CHAT-R scores and in their own diagnostic impressions were analyzed as ordinal variables. We also created a new variable called “Concordance”, where the M-CHAT-R likelihood was crossed with the PCC’s diagnostic impression, resulting into (1) concordant cases (those with High Likelihood on M-CHAT-R/diagnostic impression of Definitely Autism, and those with Low Likelihood on M-CHAT-R/diagnostic impression of Definitely Not Autism), and (2) non-concordant cases (those with High Likelihood on M-CHAT-R/diagnostic impression of Definitely Not Autism or Unsure—Needs Further Evaluation, and those with Low Likelihood on M-CHAT-R/diagnostic impression of Definitely Autism or Unsure—Needs Further Evaluation). Given that the focus was on whether concordance related to confidence and sample sizes were too small to divide into three groups (concordant, discordant, and unsure), all the Unsure—Needs Further Evaluation cases were categorized as non-concordant. We obtained descriptive statistics of (1) children with Low Likelihood and High Likelihood M-CHAT-R scores, (2) PCCs’ diagnostic impression of Definitely Not Autism, Definitely Autism, and Unsure—Needs Further Evaluation, (3) PCCs’ ratings of confidence in their diagnostic impression, (4) PCCs’ ratings of confidence on the M-CHAT-R score, and (5) type(s) of concern(s) endorsed by PCCs. Distributions of confidence ratings showed expected positive skew and some clustering at higher values, consistent with bounded Likert-type measures, without evidence of extreme ceiling effects. Due to the small sample, multi-level modeling was not feasible. Mann–Whitney U tests were used to examine (1) differences in the means of PCCs’ confidence ratings in the M-CHAT-R score by likelihood group (i.e., High Likelihood or Low Likelihood), and (2) differences in the means of PCCs’ confidence ratings in their diagnostic impressions by concordance with the M-CHAT-R outcome. We conducted a series of Chi-square tests to determine if the PCC attribution of specific concerns differed by M-CHAT-R likelihood category.

## 3. Results

Sample characteristics: Of the 50 participants, 58% of children (*n* = 29) had M-CHAT-R scores within the Low Likelihood range (score ≤ 2), and 42% (*n* = 21) within the High Likelihood range (*n*_score 3 to 7_ = 13; *n*_score≥8_ = 8). PCCs’ diagnostic impressions were Definitely Autism for 15 children (30%), Unsure—Needs Further Evaluation for 25 (50%), and “Definitely Not Autism” for 10 children (20%).

Confidence in M-CHAT-R screener score: The Mann–Whitney U test showed no significant difference between PCCs’ confidence in screener score for the High Likelihood group (M = 3.67, *SD* = 1.07) and the Low Likelihood group (M = 2.93, *SD* = 1.56), U = 214.5, *N*_HL_ = 21, *N*_LL_ = 29, *p* = 0.07, with a small to moderate effect size, *r* = 0.256.

Confidence in PCCs’ diagnostic impressions based on concordance with the M-CHAT-R autism likelihood: PCCs’ diagnostic impressions were concordant with the likelihood of autism based on the M-CHAT-R in 21 cases (42%; See Table 1). From these, nine diagnostic impressions were of Definitely Not Autism/M-CHAT-R Low Likelihood, and 12 were of Definitely Autism/M-CHAT-R High Likelihood. Among the non-concordant diagnostic impressions, three were Definitely Autism/M-CHAT-R Low Likelihood, one was Definitely Not Autism/M-CHAT-R High Likelihood, 17 were Unsure—Needs Further Evaluation/M-CHAT-R Low Likelihood, and eight were Unsure—Needs Further Evaluation/M-CHAT-R High Likelihood. The Mann–Whitney U test revealed a significant difference in their confidence in their diagnostic impression for the concordant group (M = 4.19, *SD* = 0.68) compared to the non-concordant group (M = 2.79, *SD* = 0.86), U = 70.5, *N*_conc_ = 21, *N*_non-conc_ = 29, *p* ≤ 0.001, with a large effect size, *r* = 0.677.

**Figure 1:**
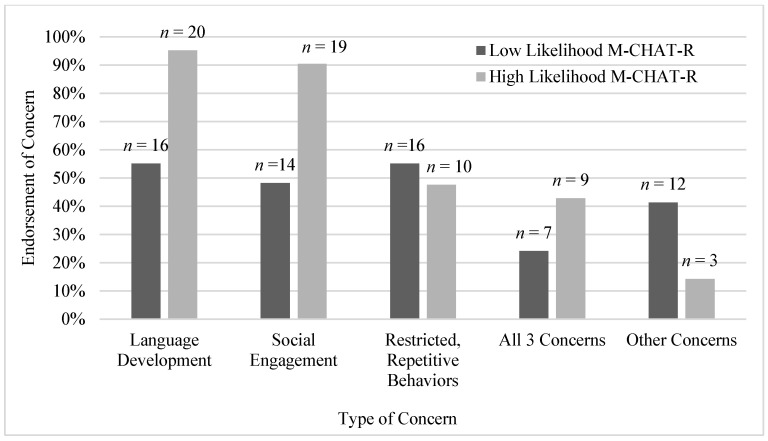
PCC’s attribution of concern leading to their diagnostic impressions (could select any number of options for each child).

PCCs’ attribution of concerns: Clinicians frequently endorsed different areas of concern for the children referred for possible autism or developmental delay (Figure 1). Language development was the most frequently endorsed concern in our sample (72%, *n* = 36), followed by social engagement (66%, *n* = 33) and restricted, repetitive behaviors (52%, *n* = 26). All three types of concerns were endorsed for 32% of children (*n* = 16). Nineteen endorsements of concerns classified as “Other” were reported across 15 children (30%), including challenging behaviors such as emotional dysregulation and head-banging (*n* = 9), gross-motor delay (*n* = 5), and sensory differences (*n* = 3). Two children were noted as “Other only”: one due to the PCC’s endorsement of teacher-reported sensory issues (*n* = 1) and one because the child was a younger sibling of a child already diagnosed with autism (*n* = 1).

We further analyzed the endorsement of each type of concern based on the autism likelihood from the M-CHAT-R score (i.e., High Likelihood vs. Low Likelihood). The frequencies of endorsement of each concern by group can be found in Table 2. The results revealed that concerns about language development and social engagement were more common among children whose M-CHAT-R score was within the High Likelihood range, whereas other types of concerns were more frequent for children with a Low Likelihood M-CHAT-R score.

## 4. Discussion

This study examined how PCCs interpret and integrate screening data from the M-CHAT-R when they have developmental concerns for a child and form an early diagnostic impression. Few studies have directly assessed clinicians’ confidence in the M-CHAT-R results, despite its widespread use in primary care to detect autism in young children (Campbell et al., 2017; Hedley et al., 2016; Robins et al., 2014). The current study helps fill this gap by quantifying clinicians’ self-reported confidence in the screener outcomes alongside their diagnostic impressions, providing new insight into how PCCs interpret autism screening results and integrate these with their clinical observations.

Consistent with the prior literature suggesting variability in concordance between screening outcomes and clinical impressions in primary care (Carbone et al., 2020; Wieckowski et al., 2025), we found that PCCs did not uniformly align their diagnostic impressions with M-CHAT-R likelihood classifications, suggesting that they were not overly reliant on the screening outcome. When concordance was present, meaning that PCCs’ impressions matched the screening outcome, PCCs reported significantly higher confidence in their diagnostic impressions. This raises the possibility that agreement between screening outcomes and clinical observation may strengthen PCCs’ certainty in their diagnostic impression decision-making, although in this study it is not possible to be certain that there is a causal relationship between concordance and confidence. Conversely, when the screening outcomes and PCCs’ diagnostic impressions were non-concordant, confidence was lower. In the majority of non-concordant cases, PCCs were unsure of their diagnostic impression; this limits interpretation, and suggests that future research should include large enough samples to examine discordance separately from non-concordance due to uncertainty.

Our results further suggest that PCCs place greater confidence in the M-CHAT-R when scores indicate a higher likelihood of autism and it aligns with their concerns for developmental delays. Parents’ reports of a child’s usual behaviors capture information across diverse contexts, which is central to the validity of early childhood autism screening tools (Robins et al., 2014). However, clinicians may vary in how much they trust or integrate these data into their diagnostic decision-making, particularly when it conflicts with their own observations and history taking during brief clinical encounters. The significant association between M-CHAT-R scores and PCCs’ confidence in the screener suggests that, for children for whom they already have developmental concerns, elevated scores bolster clinicians’ trust in the instrument whereas low scores are more often met with more reservation. In fact, reports of low referral rates in the presence of a positive screen suggest low confidence in the screener score in samples for whom the PCC does not have prior concerns (e.g., Monteiro et al., 2019). This also aligns with the literature reports of PCCs’ accuracy when ruling in autism (Ahlers et al., 2019; Penner et al., 2023; Wieckowski et al., 2025).

The distribution of diagnostic impressions also sheds light on the challenges that PCCs face when establishing a diagnostic impression for children with developmental concerns. Half of the sample was classified as Unsure—Needs Further Evaluation, suggesting that PCC uncertainty is common for children for whom PCCs have developmental concerns; this was seen both for children with Low Likelihood and those with High Likelihood screening outcomes. This hesitancy aligns with previous findings of PCCs feeling underprepared or underconfident in making diagnostic judgements about autism without specialist input (Fenikilé et al., 2015; McCormack et al., 2020). The high proportion of Unsure impressions may reflect a cautious, referral-oriented approach that needs to be studied further. However, the goal is not for PCCs to become adept at identifying and diagnosing all cases of autism, but rather for them to diagnose those cases that are more straightforward and less clinically complex. With this in mind, PCCs’ diagnosis of the 15 cases classified as “Definitely Autism” would potentially expedite entry into autism-specific early intervention and reduce the waitlist for expert evaluation to the subset of cases for whom PCCs are unsure, or M-CHAT-R scores are elevated in the absence of PCC concerns.

In order to start uncovering the factors that play a role in the process of PCCs formulating a diagnostic impression, it is helpful to consider which specific areas of development are endorsed as concerning by PCCs. The concerns most frequently endorsed were language development and social engagement, and these were endorsed more often among children with High Likelihood M-CHAT-R scores than children with Low Likelihood M-CHAT-R scores. The emphasis on language delay is consistent with previous work showing that these are often the earliest and most salient developmental concerns for both clinicians and parents (Daniels & Mandell, 2014). Fewer clinicians endorsed repetitive behaviors, which may be less evident in very young children or less commonly observed in the brief context of a primary care visit. The endorsement of all three types of concerns (language development, social engagement, and restricted repetitive behaviors) for nearly a third of the sample indicates that PCCs are attuned to the multifaceted nature of developmental delays, even when diagnostic certainty is limited, although it is interesting that the endorsement for all three concerns did not vary by M-CHAT-R likelihood. Additional concerns such as emotional dysregulation, sensory differences, and motor delays highlight the broader developmental challenges that PCCs observe and consider relevant during the diagnostic process. Several studies have shown that children’s demographics can influence both the type of concerns raised and the timing of those concerns (e.g., Donohue et al., 2019; McDonnell et al., 2021). Our study did not account for demographic differences within the children for whom specific concerns were raised and, as such, the potential association between demographic factors and the PCC’s confidence in the diagnostic impression and concerns reported remains an area that will merit further research.

Together, these findings shed light on some of the potential strengths and limitations of autism identification in primary care, while it is important to bear in mind that the present study reflects findings from a single pediatric practice. PCCs appear to recognize and act on clear cases of autism, but uncertainty remains common. When screening outcomes and clinicians’ impressions align to indicate autism and confidence is high, our findings suggest that it is appropriate for PCCs to make a diagnostic determination, consistent with previous reports (McNally Keehn et al., 2023; Penner et al., 2023). This will help some children access autism-specific early intervention more rapidly. It also may reduce the waiting time for those children who need to be seen by a specialist to determine if autism is an appropriate diagnosis or not. In contrast, when the screening outcome and the diagnostic impressions are non-concordant, or when confidence in their diagnostic impression is low, PCCs should refer children to experts who can conduct an in-depth diagnostic evaluation for autism. Although this is an important step in these cases, it will likely delay the diagnostic process for some children. Frequent PCC uncertainty about their own diagnostic impression further underscores the need for supported decision-making systems to increase accuracy and self-efficacy, for example, through access to brief teleconsultation with developmental specialists, integrated behavioral health models within the primary care system, training and consulting for PCCs (e.g., Bickel et al., 2025; Guan et al., 2022; Sohl et al., 2022), and training PCCs to administer certain autism diagnostic tools and develop independent competency in autism evaluation. Examples of the latter are the STAT MD (McNally Keehn et al., 2024), an adaptation of the training to use the Screening Tool for Autism in Toddlers and Young Children and conduct autism-focused assessments in pediatric clinics, or the ASD-PEDS, a free tool developed for the observation of autism-related behaviors in toddlers and young children that takes 15 to 30 min to implement (Hine et al., 2023). While this type of training might not be feasible for all PCCs, it may help provide a more sustainable pathway to autism services, given that many service providers and insurers still refuse to acknowledge autism diagnoses without formal testing.

Several limitations of the current study should be noted. The small sample size of PCCs and children and the single-site design limit generalizability, including the inability to conduct multi-level analysis of PCCs’ ratings and the need to combine discordant impressions with those that were non-concordant due to uncertainty. Characteristics of the clinic, such as clinician experience or prior autism training, may have influenced confidence ratings, yet were not collected as part of this study. Missing M-CHAT-R data for 10 participants within the age range for receiving the M-CHAT-R may have introduced bias. Additional potential contextual influences on PCCs’ confidence ratings, such as time constraints during visits, parental-initiated concern, or family’s prior exposure to autism diagnosis or developmental concerns, were not measured or controlled for in this study. Lastly, the psychometric limitations of Likert scales include response biases (central tendency, acquiescence, social desirability), the assumption of equidistant intervals (which is not always true), and unidimensionality (not capturing complex attitudes), all potentially affecting true attitude measurement and data interpretation (Sullivan & Artino, 2013). Future studies with larger, more diverse samples could allow finer-grained analysis of how confidence relates to diagnostic accuracy and would allow for more sophisticated analyses accounting for the nesting of children within PCCs.

Future work might also investigate further differences in PCCs’ confidence on their diagnostic impressions among the cases we addressed as “non-concordant” in this study (i.e., either removing the option to select of “Unsure—Needs Further Evaluation” or examining these cases separately from discordant cases) and longitudinal outcomes for children diagnosed in primary care versus specialty settings, as well as demographic or system-level predictors of clinician confidence. Exploring parent–clinician communication and shared decision-making during early evaluations may further clarify how screening information translates into timely intervention.

## 5. Conclusions

Primary care clinicians play a pivotal role in the early detection of autism. Our findings suggest that PCCs may already appropriately integrate screening data with clinical judgment, demonstrating confidence in clear cases while exercising caution in less clear ones. Strengthening PCC training and providing structured consultation pathways, both within and outside of primary care settings, could enhance diagnostic confidence, potentially streamline referrals, and ultimately reduce delays in accessing evidence-based intervention.

## Figures and Tables

**Table 1:** PCCs’ diagnostic impression and concordance with M-CHAT-R outcome.

	Low Likelihood M-CHAT-R (Score ≤ 2) *n* = 29	High Likelihood M-CHAT-R (Score ≥ 3) *n* = 21
Definitely Autism	3 (6%)	**12 (24%)**
Definitely Not Autism	**9 (18%)**	1 (2%)
Unsure—Needs Further Evaluation	17 (34%)	8 (16%)

Bolding indicates cases labeled as “Concordant” for Mann–Whitney U test.

**Table 2:** Frequency of PCC-endorsed concerns by M-CHAT-R autism likelihood.

Type of Concern	Low Likelihood M-CHAT-R (Score ≤ 2) *n* = 29	High Likelihood M-CHAT-R (Score ≥ 3) *n* = 21	χ^2^ (df, N)	*p*
Language Development	16 (55.1%)	20 (95.2%)	9.69 (1, N = 36)	0.002
Social Engagement	14 (48.2%)	19 (90.4%)	9.66 (1, N = 33)	0.002
Restricted, Repetitive Behaviors	16 (55.1%)	10 (47.6%)	0.27 (1, N = 26)	0.598
All 3 Concerns	7 (24.1%)	9 (42.8%)	1.96 (1, N = 16)	0.161
Other Concerns	12 (41.3%)	3 (14.2%)	4.25 (1, N = 15)	0.039

## Data Availability

Data from this study are not part of any public repository.
